# The Diversity of Spotted Fever Group *Rickettsia* Found in *Ixodidae* Hard Ticks Removed from Humans in Karachay-Cherkessia, North Caucasus, Russia

**DOI:** 10.3390/microorganisms12071293

**Published:** 2024-06-25

**Authors:** Alexey V. Rakov, Tatiana A. Chekanova, Ketevan Petremgvdlishvili, Svetlana B. Linnik, Khusey Kh. Batchaev, Vasiliy G. Akimkin

**Affiliations:** 1 Laboratory for Natural Focal Infections Epidemiology, Central Research Institute of Epidemiology, 111123 Moscow, Russia; tchekanova74@mail.ru (T.A.C.); ketevan0511@mail.ru (K.P.); 2 Center of Hygiene and Epidemiology in the Karachay-Cherkess Republic, 369000 Cherkessk, Russia; splyasunova@mail.ru (S.B.L.); husey.batchaev@mail.ru (K.K.B.); 3 Central Research Institute of Epidemiology, 111123 Moscow, Russia; vgakimkin@yandex.ru

**Keywords:** ticks, *Rickettsia*, North Caucasus, *Borrelia*, *Anaplasma*

## Abstract

Karachay-Cherkessia is the region in the Russian Federation that has been overlooked in terms of research on the human bacterial pathogens transmitted by ticks. In this study, we investigated the infection status of ticks with *Rickettsia*, *Borrelia*, *Coxiella burmetii*, *Anaplasma phagocytophilum*, and *Ehrlichia chaffeensis*/*Ehrlichia muris* associated with natural focal infections in Karachay-Cherkessia. A total amount of 159 ticks from three species across three genera (*Ixodes ricinus*, *Dermacentor marginatus*, *Hyalomma scupense*) removed from humans were collected for analysis. Additionally, we used 53 individual ticks and 40 tick pools from the vegetation of three species (*I. ricinus*, *D. marginatus*, and *Rhipicephalus bursa*). Screening of the studied pathogens was performed by using commercial qPCR kits. Sanger sequencing utilizing partial sequences of *gltA* and *ompA* genes was employed to identify the *Rickettsia* species. Our findings revealed the presence of DNA from five species within the spotted fever group *Rickettsia*, namely *Rickettsia raoultii*, *R. slovaca*, *R. helvetica*, *R. monacensis*, and *R. aeschlimannii*. Moreover, two distinct genotypes were identified within *R. aeschlimannii*, *R. helvetica*, and *R. monacensis*. Additionally, DNA from *Borrelia burgdoferi* s.l., *B. miyamotoi*, and *A. phagocytophilum* was detected in the tested ticks. This study provides valuable insights into the prevalence and the diversity of bacterial pathogens transmitted by ticks in the Karachay-Cherkessia region.

## 1. Introduction

In the Russian Federation, the official registration of infectious diseases is carried out by Rospotrebnadzor, which is the main agency with the function of sanitary and epidemiological supervision. Vector-borne human infections are monitored by Sanitary Epidemiological Surveillance Agencies and include tick-borne encephalitis, Lyme disease, Congo-Crimean hemorrhagic fever (CCHF), Omsk hemorrhagic fever (OHF), Rickettsioses (including North Asian tick typhus (NATT, synonym: Siberian tick typhus) and Astrakhan spotted fever (ASF)), human monocytic ehrlichiosis (HME), human granulocytic anaplasmosis (HGA), tularemia, and Q fever. The State Veterinary Service is responsible for the monitoring of only five animal tick-borne diseases: bovine babesiosis, theileriosis, bovine anaplasmosis, equine babesiosis, and equine nuttalliosis [1]. The spectrum of pathogens, which are in the monitoring by these agencies, differs. Many tick-borne micro-organisms are not monitored by government services. As a result, many infectious diseases carried by ticks, including rickettsioses of the spotted fever group *Rickettsia* (SFGR)—which do not cause the above-mentioned nosologies (NATT and ASF)—remain unrecognized. At the same time, the count of individuals bitten by ticks seeking medical attention in the Russian Federation is also documented.

The European south of the Russian Federation is known as an endemic region for the number of the natural focal infections, such as CCHF [2], West Nile fever (WNF) [3], Q fever [4], and ASF [3]. Much less attention has been paid to other infections such as tick-borne rickettsiosis and borreliosis.

**Figure 1:**
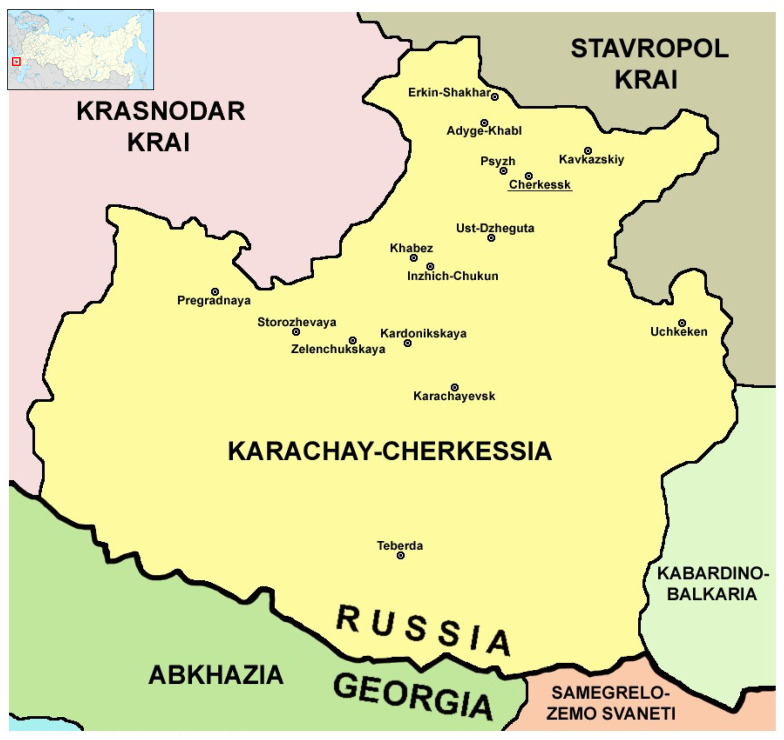
Administrative map of Karachay-Cherkessia with major settlements and neighboring regions (modified from https://commons.wikimedia.org/wiki/File:Karachay_Cherkess03.png; accessed on 4 April 2024). The location on the map of Russia is displayed in the top left corner.

The Karachay-Cherkessia (Karachay-Cherkess Republic) is a region in Russia located in the North Caucasus on the border of Europe and Asia. It is bordered by Krasnodar Krai to the west, Stavropol Krai to the north, Kabardino-Balkaria to the east, and Georgia to the south (Figure 1). The capital city is Cherkessk (the population is about of 112,000), and the total population of the republic is approximately 470,000. Due to the development of domestic tourism in the Russian Federation in recent years, the region has shown one of the highest growth rates of domestic tourism. The most important direction of the tourism in the republic is the ski resorts. The mountainous areas of the Arkhyz and Dombay resorts with developed infrastructure are the most famous tourist attractions. Mount Elbrus is on the border of Karachay-Cherkessia, and Kabardino-Balkaria is the highest mountain peak in Europe and Russia, which also attracts many adventure enthusiasts [5].

In the fauna of the North Caucasus, 38 species of ticks of five genera have been recorded. At the same time, the most common are *Ixodes ricinus* (Linnaeus, 1758), *Haemophysalis punctate* (Canestrini et Fanzago, 1878), *Dermacentor reticulatus* (Fabricius, 1794), *Dermacentor marginatus* (Sulzer, 1776), *Rhipicephalus rossicus* (Jakimov et Kohl-Jakimova, 1911), *Hyalomma marginatum* (Koch, 1844), and *Hyalomma scupense* (Schulze, 1919) [6].

Despite the considerable diversity of ixodofauna in the North Caucasus, the level of knowledge about their infectivity leaves much to be desired. The most-studied region within the North Caucasian Federal District is the Stavropol Krai region [7]. The highest incidence of SFGR in the districts of Stavropol Krai has been reported to range from 44.3 to 58.8% [7]. In contrast, the Karachay-Cherkessia Republic remains relatively underexplored in this regard, with only a few publications being available, which are primarily in the form of abstracts from local scientific conferences.

Based on the public data from the state reports of Rospotrebnadzor [8,9], it was observed that in 2021 and 2022, 235 and 136 individuals, respectively, were documented as having experienced tick bites. The dominant species attacking humans were identified as *I. ricinus* and *D. marginatus*. In 2022, a total of 59 tick pools were tested using PCR. Notably, no CCHF virus RNA was detected in the analyzed samples. However, *Borrelia* DNA was identified in three pools of *I. ricinus* from Cherkessk, the Adyge-Khabl district, and Novo-Kuvinsk. Furthermore, the DNA of SFGR was detected in 21 *D. marginatus* samples across various locations: Cherkessk city (eight), the Prikubansky district (nine), the Abazinsky district (one), the Malokarachayevsky district (one), and the Adyge-Khablsky district (two). Species identification was not conducted in this study.

Most of the available studies have relied on the analysis of field material (ticks) in pools, which has its own limitations. Specifically, this approach often gives controversial results, and the calculation of the real infection rate (the MIR or minimum infection rate) is biased [10].

This work is the study of the presence of bacterial natural focal infection pathogens in individual ticks, which has been conducted for a more accurate understanding of the tick-borne infection in the region. The pools can contain DNA of different species of the same pathogen and subsequently have an ambiguous interpretation regarding the Sanger sequencing results. Taking all of the above into consideration, there is a high probability that there is an underdiagnosis of tick-borne bacterial infections in this region. Our objective was to ascertain, for the first time, the spectrum of bacterial infections transmitted by ticks by identifying the pathogen species present in the Karachay-Cherkessia Republic. The findings will assist in improving the precision and efficacy of public health diagnostic endeavors in the examined region.

The aim of this study is to investigate the prevalence of the known bacterial pathogens in *Ixodidae* ticks removed from outpatients in Karachay-Cherkessia, particularly the spotted fever groups *Rickettsia*, *Borrelia*, *Coxiella burnetii, Anaplasma phagocytophilum*, and *Ehrlichia chaffeensis/Ehrlichia muris*, to determine the genospecies diversity of tick-borne SFGR.

## 2. Materials and Methods

### 2.1. Study Area

Flora: The Karachay-Cherkessia Republic is located in mountain steppe and deciduous forest zones. The flora and fauna of Karachay-Cherkessia, as well as of the entire Caucasus, are extremely diverse due to the altitudinal zonation. Caucasus flora is one of the richest in these latitudes of the globe, including at least 6350 species. Forests are one of the main components of the landscape of both the plain and mountainous regions of Karachay-Cherkessia, above which there are subalpine and alpine meadows, which are valuable mountain pastures. Subalpine meadows follow alpine meadows with three types of vegetation: low grassy meadows, scree vegetation, and rocky vegetation.

Climate: The climate is mild and moderately warm, which attracts tourists to the area throughout the year. The winters are short, with average winter temperatures as low as −5 °C in the plains and −10 °C in the mountains. Snowfall there is quite heavy. In the middle of a long summer, the air heats up to +20 °C, sometimes to +29 °C, and even to +39 °C in the plains and to +8 °C in the highland. The average temperature in January is −10.2 °C and in July is +22.6 °C. An abundant amount of rainfall is observed in the region throughout the year [11].

### 2.2. Tick Collection

A total of 159 ticks of 3 species was removed from humans between the period from April to September 2021: *I. ricinus* (117), *D. marginatus* (35), and *Hy. scupense* (5) were analyzed. In addition, 53 ticks of three species collected from vegetation in the period of April to May 2021 were characterized: *I. ricinus* (5), *D. marginatus* (40), and *R. bursa* (8). Additionally, ticks of three species collected from vegetation in April 2020, *I. ricinus* (1), *D. marginatus* (34), and *R. bursa* (5), which were analyzed in 40 pools (Table S1). Ticks were morphologically identified according to the method described by Filippova [12].

The majority of tested ticks (70.5%) were from Cherkessk at 96 (60.4%), and Karachayevsk at 16 (10.1%). From the remaining settlements in the Abazinsky, Karachayevsky, Nogaysky, and Ust-Dzhegutsky districts (auls, urban-type settlements, villages, and stanitsas) 47 ticks (29.5%) were collected from 1 to 4 ticks per settlement. Ticks collected from vegetation were found in the Abazinsky, Adyge-Khablsky, Nogaysky, Prikubansky, and Khabezsky districts, as well as in the city of Cherkessk (Figure S1).

Ticks were collected from vegetation using flagging method. Briefly, ticks were collected in the daylight hours by dragging a flag 1.5 × 2.0 m in size over vegetation in the abovementioned zones. All ticks were starved (unfed). Ticks attached to the flag were removed, placed into individual Eppendorf tubes, and stored at −70 °C until transportation to the laboratory. Transportation to the laboratory was carried out by air in a thermal container with enclosed ice packs for 3 days. Homogenization and DNA extraction were carried out within a week from the day that they arrived at the laboratory. The isolated DNA and the remains of the homogenates were stored at −20 °C during the month when PCR and sequencing were performed. Subsequently, all nucleic acid residues and homogenates were transferred for long-term storage at −70 °C.

### 2.3. DNA Extraction and Quantitative PCR

Each tick was individually washed with 96% ethanol and then 0.15 M NaCl solution. Ticks were homogenized in a 2.0 mL Eppendorf tube in 300 µL 0.15 M NaCl solution with tungsten carbide beads in a TissueLyser LT homogenizer (Qiagen, Hilden, Germany) at 50 Hz/s for 10 min. Total DNA was extracted using the «AmpliSens^®^ RIBO-prep» kit (CRIE, Moscow, Russia). The qPCR screening was performed: for *Rickettsia* spp. using the «AmpliSens^®^ *Rickettsia* spp. SFG-FL» kit targeting the *ompB* gene, for *C. burnetii* using the «AmpliSens^®^ *Coxiella burnetii*-FL» kit, for *Borrelia miyamotoi* using the «AmpliSens^®^ *Borrelia miyamotoi*-FL» kit targeting the *glpQ* gene, for TBEV, *Ehrlichia chaffeensis*/*E. muris*, *Anaplasma phagocytophillum*, and *Borrelia burgdorferi* sensu lato using the «AmpliSens^®^ TBEV, *B. burgdorferi* sl, *A. phagocytophillum*, *E. chaffeensis*/*E. muris*-FL» kit according to the manufacturer’s instructions. All kits are manufactured by the CRIE, Moscow, Russia. The Rotor-Gene Q qPCR cycler (Qiagen, Hilden, Germany) was used.

### 2.4. Conventional PCR and Sanger Sequencing

The genospecies of SFGR were determined by Sanger sequencing of the citrate synthase *gltA* (382 bp) and outer membrane protein A *ompA* (532 bp) partial genes using both DNA strands with appropriate primers after PCR amplification [13]. BLASTN 2.15.0 with default parameters used to search for homologous sequences in the GenBank nr/nt database. The genotype of *B. miyamotoi* was determined using the partial sequence of the antitermination transcription factor *nusB* gene (387 bp) [14].

### 2.5. Phylogenetic Analysis

Dendrograms were constructed in MEGA 6.06 using the maximum likelihood on aligned sequences of both genes with a bootstrap value of 1000. Homologous DNA sequences from the complete genomes of the corresponding representative SFGR obtained from GenBank were used for comparison. A homologous fragment of the *R. bellii* An04 genome sequence (NZ_CP015010) was used as an outgroup to construct a dendrogram using the *gltA* partial gene sequence.

The modified Wald method was used in QuickCalcs (GraphPad, San Diego, CA, USA) to calculate 95% confidence intervals (CI) of tick infection rates.

The sequences from this study are available in GenBank (PP431024-PP431074, OR192576).

## 3. Results

The DNA amounts of SFGR were detected using qPCR screening in 31 ticks (n = 24 *I. ricinus* ticks, n = 6 *D. marginatus* ticks, and n = 1 *Hy. scupense*) removed from humans (Table 1). *Rickettsia* species identification was performed by sequencing the *gltA* gene fragment for all positive samples. The rickettsial DNA from 7 ticks removed from humans was not analyzed using Sanger sequencing due to the low concentration of qPCR amplicons. The *ompA* gene fragment sequencing was used for the confirmation of additional species in some samples. The results of the SFGR genospecies typing are summarized in Table 1. The SFG *Rickettsia* of five species, *R. monacensis* (10), *R. helvetica* (6), *R. raoultii* (synonym: *R. conorii* subsp. *raoultii*) (3), *R. aeschlimannii* (3), and *R. slovaca* (2), have been detected in ticks removed from humans. Nine tick specimens (n = 8 *D. marginatus* and n = 1 *I. ricinus*) collected from vegetation in 2021 were positive for *R. raoultii* (7), *R. slovaca* (1), and *R. helvetica* (1) (Table 2), and six *D. marginatus* tick pools collected from vegetation in 2020 were positive for *R. raoultii* (5) and *R. slovaca* (1) (Table 3).

Thus, ticks collected from vegetation were dominated by *R. raoultii* (13.2%, 6.2–25.1% 95% CI), while ticks removed from humans were dominated by *R. monacensis* (6.3%, 3.3–11.3% 95% CI).

**Figure 2:**
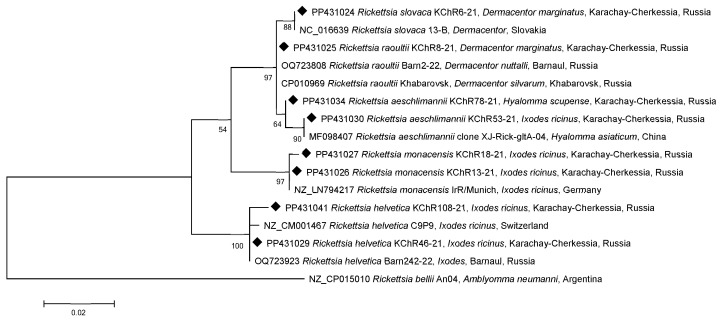
Phylogenetic tree constructed using the maximum likelihood method based on nucleotide sequences of *Rickettsia* spp. from ticks, including ones from this study (Karachay-Cherkessia, black diamonds) and reference sequences of the *gltA* gene fragment (384 bp). The *R. bellii* An04 (NZ_CP015010) sequence was used as an outgroup. The GenBank accession numbers for reference sequences are shown with the sequence name, tick species, and country. The branch numbers indicate bootstrap support (1000 replicates). The scale bar indicates the phylogenetic distance.

**Figure 3:**
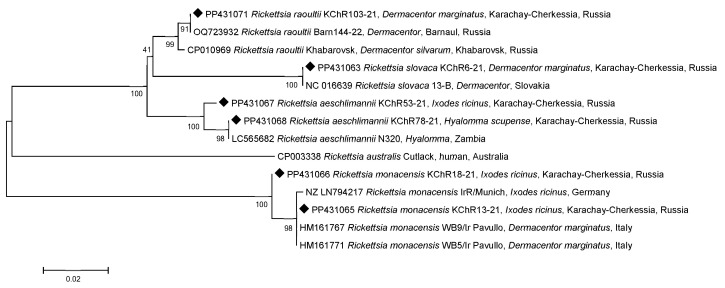
Phylogenetic tree constructed using the maximum likelihood method based on nucleotide sequences of *Rickettsia* spp. from ticks, including ones from this study (Karachay-Cherkessia, black diamonds) and reference sequences of the *ompA* gene fragment (532 bp). The GenBank accession numbers for reference sequences are shown with the sequence name, tick species, and country. The branch numbers indicate bootstrap support (1000 replicates). The scale bar indicates the phylogenetic distance.

All isolated and sequenced DNA samples of *R. raoultii* belonged to the same RpA4 genotype according to fragments of both *gltA* (15 samples) and *ompA* (5 samples) genes [15] (Figure 2 and Figure 3).

Similarly, all four samples of *R. slovaca* were isolated from *D. marginatus* and were identical to each other for both genes and to the reference genome 13-B (NC_016639) from Slovakia (Figure 2 and Figure 3).

All ten *R. monacensis* DNAs were isolated from ticks that had been removed from humans. Interestingly, one tick of ten belonged to *D. marginatus*, while nine belonged to *I. ricinus*. One sample of *R. monacensis* KChR18-21 from *I. ricinus* differed from the other nine by one substitution in the *gltA* gene fragment (Figure 2) and four substitutions in the *ompA* gene fragment (Figure 3). Based on the *gltA* gene fragment, similar clones in the GenBank database were not identified as *R. monacensis* and were the same as those from Slovakia (*I. ricinus*, AF140706), Portugal (*I. ricinus*, EF501755), and China (*I. sinensis*, OP125489, OP125492). The remaining nine *R. monacensis* were 100% identical to the reference strain IrR/Munich (LN794217) in fragments of both genes (Figure 2). Four of the ten *R. monacensis* were further sequenced for the partial *ompA* gene: three were 100% identical to samples from Italy (*D. marginatus*, HM161767, HM161771) and Serbia (*I. ricinus*, GQ925821) (Figure 3). Although the main reservoir for *R. monacensis* is *I. ricinus*, it is sometimes found in *D. marginatus*. Moreover, the *R. monacensis* KChR18-21 from *I. ricinus* was identical to another sample from Italy, as well as also isolated from *I. ricinus* (HM161773). It differs from the IrR/Munich reference genome by five substitutions.

DNA from *R. helvetica* has been detected in six samples of *I. ricinus* that were removed from humans. Another DNA of *R. helvetica* was detected in *I. ricinus* collected from vegetation (Cherkessk) in the same year 2021 and belonged to the same genotype. This genotype differed from the reference genome C9P9 (NZ_CM001467) by a single substitution in a fragment of the *gltA* gene and was identical only to the *R. helvetica* that we had previously studied in Barnaul, Western Siberia, Russia (Figure 2) [12]. A DNA sample from *R. helvetica* KChR108-21 differed from the reference genome by a deletion at position 6 in addition to this substitution in the *gltA* gene. These samples are not present in the dendrogram (Figure 3), as *R. helvetica* is characterized by the absence of the *ompA* gene.

Two DNA samples of *R. aeschlimannii* by the *gltA* gene fragment were 100% identical to partial genomes from the GenBank isolated in Russia (Tyumen Oblast, Republic of Kalmykia), China, Iran, Egypt, Mauritania, and Senegal. However, DNA sample KChR53-21 differed from them by two nucleotides and was identical to the clone XJ-Rick-gltA-04 of *Hy. asiaticum* from China (MF098407) (Figure 2) and differed from the closest genome of *R. aeschlimannii* N320 (LC565682) from the Zambian tick of the genus *Hyalomma* by seven substitutions in the *ompA* gene (Figure 3).

In addition to studying the species diversity of SFGR, screening tests showed that the infection rate of *I. ricinus* by *B. burgdorferi* s.l. was 5.1% (95% CI 2.1–11.0%), and that of *D. marginatus* was 2.8% (95% CI 0–15.8%) (Table 4). Single specimens were also found in ticks that were collected from the vegetation (Table 5 and Table 6).

Two DNA samples of *B. miyamotoi* (1.7%, 95% CI 0–6.4%) isolated from *I. ricinus* removed from humans were assigned to clonal complex II, including the European sequence type ST-10 and the new sequence type ST-11, using multilocus sequencing typing, and they differed significantly from the other *B. miyamotoi* found in ticks on the territory of the Russian Federation belonging to clonal complex I [14]. *B. miyamotoi* of sequence type ST-10 has previously been found in the Czech Republic and in the Netherlands.

Furthermore, there was a single DNA detection of the human granulocytic anaplasmosis causative agent *A. phagocytophillum* in one of the *I. ricinus* ticks (0.85%, 95% CI 0–5.2%) (Table 4). In none of the ticks tested was DNA detected from *C. burnetii* nor the human monocytic ehrlichiosis causative agents *E. chaffeensis* and *E. muris*.

Only two cases of coinfection of *I. ricinus* ticks were detected: triple *B. burgdorferi* s.l., *B. miyamotoi* and *A. phagocytophillum* were detected in one tick, and double *Rickettsia* spp. and *B. miyamotoi* were detected in the other.

## 4. Discussion

It is known that the habitat of ticks of certain genera is limited to the features of the landscape of the biotope. For example, *Dermacentor* ticks predominantly inhabit grassland communities, while *Ixodes* ticks prefer wooded habitats [16]. In the case that their habitats may overlap, it is possible to encounter most of the characteristic pathogens of one tick genus and the members of another genus in neighboring ticks. The majority of ticks examined in our study (95.6%) were identified as belonging to the two species *I. ricinus* and *D. marginatus*.

The highest level of *I. ricinus* and *D. marginatus* infection was noted for tick-borne SFGR pathogens. The overall infection rate for *I. ricinus* was 20.5%, and the genospecies structure of the SFGR was the most diverse, with four out of the five genospecies detected. In comparison, the infection rate and the diversity of *Rickettsia* species in *D. marginatus* ticks were slightly lower at 17.1% and three genospecies, respectively. At the same time, in addition to the revealed sympatry, the associations of certain host and pathogen species were observed: *I. ricinus* showed association with *R. helvetica* and *R. monacensis*, while *D. marginatus* was associated with *R. raoultii* and *R. slovaca*. These findings align with the already well-known data previously reported in other parts of the world, as well as in Russia [13,17,18].

It is known that *R. raoultii* genotype Rp4 has been identified and is dominant in Europe, as well as in the European and Asian parts of Russia up to Eastern Siberia [18,19]. The same genotype has already been identified in the Altai Krai, Western Siberia [13] (Figure 2 and Figure 3). A comparison of partial genes from the GenBank database revealed that *R. slovaca* exhibited identical sequences to those from Europe (Slovakia), and *R. monacensis* showed relations to strains found in Europe (Italy, Portugal, Slovakia, and Serbia) and China. In contrast, *R. aeschlimannii* was found to be closely related to samples from Asia (China and Iran) and Africa (Egypt, Mauritania, Senegal, and Zambia). We can infer that the range of these rickettsial genotypes is expanding due to the migration of vectors carrying the identified SFGR species, which is facilitated by the broadening habitat of animals and birds that serve as hosts for the ticks moving from south to north [20].

The difference in the prevalence of *R. monacensis* in *I. ricinus* ticks collected from humans and *R. raoultii* in *D. marginatus* ticks collected from vegetation can be attributed to the distinct habitat of these species [16]. There was a significantly lower rate of infection with other pathogens. Furthermore, mixed infections were also uncommon in our sampling, with only two *I. ricinus* tick samples exhibiting triple and double coinfections.

Among the regions bordering on Karachay-Cherkessia in the Russian Federation, Stavropol Krai has been extensively researched in this context. A study of 824 tick pools collected in 2016 in Stavropol Krai, which borders Karachay-Cherkessia in the south, revealed the presence of *Rickettsia* DNA from five species: *R. massiliae* (11 samples), *R. raoultii* (4), *R. sibirica* (2), *R. aeschlimannii* (2), and *R. slovaca* (1) [6]. Further examination of the tick pools of *Hy. marginatum*, *D. marginatus*, *D. reticulatus*, *Ha. punctata*, *I. ricinus*, *R. rossicus*, and *R. turanicus* collected between 2017 and 2021 identified five SFGR species: *R. raoultii* (24 samples), *R. aeschlimannii* (12), *R. slovaca* (10), *R. massiliae* (2), and *R. helvetica* (1) [21]. The SFGR genospecies were not identified from clinical samples [7]. Due to the pooled nature of the studies, the tick infection rates were not calculated. In contrast to the above data, we identified 10 DNA specimens of *R. monacensis* in ticks removed from patients in Karachay-Cherkessia, which were not found in Stavropol Krai. We did not detect this *Rickettsia* species in ticks collected from vegetation, which can be explained by the fact that we mainly collected *D. marginatus* ticks from vegetation (79.6%) rather than *I. ricinus* ticks (6.4%), where the overwhelming majority of *R. monacensis* was found.

Nine SFGR genospecies were found in the neighboring north of Karachay-Cherkessia in the country of Georgia: *R. conorii*, *R. aeschlimannii*, *R. raoultii*, *R. slovaca*, *R. massiliae*, *R. monacensis*, *R. helvetica*, *R. hoogstraalii*, and *Candidatus* R. barbariae [22]. The study used 1594 individual ticks from five genera—both pooled and unpooled. Thus, five of the nine species were also found in Karachay-Cherkessia.

Although *R. aeschlimannii* is known to persist primarily in ticks of the genus *Hyalomma*, two of the three detections in our study were in *I. ricinus* ticks. This is the first report of the detection of *R. aeschlimannii* in the tick species *Hy. scupense* in Russia. Interestingly, *R. aeschlimannii* was first detected in Russia in 2004 in *Hy. Marginatum* in Stavropol Krai [23]. Moreover, *R. aeschlimannii* has been identified in *Hy. scupense* in Georgia, which is also on the border of Karachay-Cherkessia [22]. Our data are also consistent with the other results obtained in Georgia: the main vector for *R. raoultii* and *R. slovaca* is *D. marginatus*, for *R. helvetica* and *R. monacensis* is *I. ricinus*, and for *R. aeschlimannii* is *Hy. scupense* (Table 1).

The data presented on the comparison of the partial nucleotide sequences of the citrate synthase and outer membrane protein A genes indicate that the *R. raoultii* and *R. slovaca* found in *D. marginatus* belong to relatively genetically conservative species, whereas the *R. helvetica*, *R. monacensis*, and *R. aeschlimannii* belong to more diverse species, since two genotypes were identified for each genospecies, some of which were observed for the first time.

In recent years, clinical cases of tick-borne rickettsiosis caused by pathogens identified in the present study, namely *R. raoultii* [24], *R. aeschlimannii*, and *R. slovaca* [25], have been reported in the Russian Federation. While diseases associated with *R. helvetica* and *R. monacensis* have not yet been confirmed in Russia, their association with the clinical manifestations has been identified in European countries: *R. helvetica* in Sweden [26] and *R. monacensis* in Spain and Italy, and the disease was similar to the clinic of Mediterranean spotted fever [27,28]. Obviously, the diagnosis of infections caused by these pathogens is challenging.

Thus, in the present study, we revealed for the first time a significant species diversity of SFGR in Karachay-Cherkessia compared to other regions of the Russian Federation. The detected species also had a genetic diversity in the *gltA* and *ompA* gene fragments that were sequenced. It is necessary to continue and to expand the study of the population structure of SFGR in ticks of different species across the North Caucasus. This will help to substantiate the content and the scope of the diagnostic and preventive measures in these Russian regions.

## Figures and Tables

**Table 1:** Prevalence of tick-borne rickettsioses pathogens in ticks removed from humans in Karachay-Cherkessia in 2021.

Tick Species	Number of Ticks	Number of Ticks Infected by SFGR (%, 95% CI)						
		*R. raoultii*	*R. helvetica*	*R. monacensis*	*R. slovaca*	*R. aeschlimannii*	Not Studied	Total *Rickettsia* spp.
*I. ricinus*	117	1 (0.85%, 0–5.2%)	6 (5.1%, 2.1–11.0%)	9 (7.7%, 4.0–14.1%)	0	2 (1.7%, 0–6.4%)	6 (5.1%, 2.1–11.0%)	24 (20.5%, 14.1–28.8%)
*D. marginatus*	35	2 (5.7%, 0–20.0%)	0	1 (2.8%, 0–15.8%)	2 (5.7%, 0–20.0%)	0	1 (2.8%, 0–15.8%)	6 (17.1%, 7.7–33.1%)
*Hy* *. scupense*	5	0	0	0	0	1 (20.0%, 2.0–64.0%)	0	1 (20.0%, 2.0–64.0%)
Unidentified	2	0	0	0	0	0	0	0
Total	159	3 (1.9%, 0–5.6%)	6 (3.8%, 1.6–8.2%)	10 (6.3%, 3.3–11.3%)	2 (1.3%, 0–4.8%)	3 (1.9%, 0–5.6%)	7 (4.4%, 2.0–9.0%)	31 (19.5%, 14.0–26.4%)

**Table 2:** Prevalence of tick-borne rickettsioses pathogens in ticks collected from vegetation in Karachay-Cherkessia in 2021.

Tick Species	Number of Ticks	Number of Ticks Infected by SFGR (%, 95% CI)			
		*R. raoultii*	*R. helvetica*	*R. slovaca*	Total *Rickettsia* spp.
*I. ricinus*	5	0	1 (20.0%, 2.0–64.0%)	0	1 (20.0%, 2.0–64.0%)
*D. marginatus*	40	7 (17.5%, 8.4–32.3%)	0	1 (2.5%, 0–14.0%)	8 (20.0%, 10.2–35.0%)
*R. bursa*	8	0	0	0	0
Total	53	7 (13.2%, 6.2–25.1%)	1 (1.9%, 0–10.9%)	1 (1.9%, 0–10.9%)	9 (17.0%, 9.0–29.4%)

**Table 3:** Prevalence of tick-borne rickettsioses pathogens in tick pools collected from vegetation in Karachay-Cherkessia in 2020.

Tick Species	Number of Tick Pools	Number of Tick Pools Infected by SFGR (%, 95% CI)		
		*R. raoultii*	*R. slovaca*	Total *Rickettsia* spp.
*I. ricinus*	1	0	0	0
*D. marginatus*	34	5 (14.7%, 6.0–30.6%)	1 (2.9%, 0–16.2%)	6 (17.6%, 8.0–33.9%)
*R. bursa*	5	0	0	0
Total	40	5 (12.5%, 5.0–26.6%)	1 (2.5%, 0–14.0%)	6 (15.0%, 6.7–29.5%)

**Table 4:** Tick-borne pathogens whose DNA was detected in ticks removed from humans in Karachay-Cherkessia in 2021.

Tick Species	Number of Ticks	Number of Ticks Infected by (%, 95% CI)				
		*B. burgdoferi* s.l.	*B. miyamotoi*	*C. burnetii*	*A. phagocytophillum*	*E. chaffeensis/E. muris*
*I. ricinus*	117	6 (5.1%, 2.1–11.0%)	2 (1.7%, 0–6.4%)	0	1 (0.85%, 0–5.2%)	0
*D. marginatus*	35	1 (2.8%, 0–15.8%)	0	0	0	0
*Hy. scupense*	5	0	0	0	0	0
Unidentified	2	0	0	0	0	0
Total	159	7 (4.4%, 2.0–9.0%)	2 (1.3%, 0–4.8%)	0	1 (0.63%, 0–3.8%)	0

**Table 5:** Tick-borne pathogens whose DNA was detected in ticks collected from vegetation in Karachay-Cherkessia in 2021.

Tick Species	Number of Ticks	Number of Ticks Infected by (%, 95% CI)				
		*B. burgdoferi* s.l.	*B. miyamotoi*	*C. burnetii*	*A. phagocytophillum*	*E. chaffeensis/E. muris*
*I. ricinus*	5	1 (20.0%, 2.0–64.0%)	0	0	0	0
*D. marginatus*	40	0	0	0	0	0
*R. bursa*	8	0	0	0	0	0
Total	53	1 (1.9%, 0–10.8%)	0	0	0	0

**Table 6:** Tick-borne pathogens whose DNA was detected in tick pools collected from vegetation in Karachay-Cherkessia in 2020.

Tick Species	Number of Tick Pools	Number of Tick Pools Infected by (%, 95% CI)				
		*B. burgdoferi* s.l.	*B. miyamotoi*	*C. burnetii*	*A. phagocytophillum*	*E. chaffeensis/E. muris*
*I. ricinus*	1	1 (100.0%, 16.7–100.0%)	0	0	0	0
*D. marginatus*	34	0	0	0	0	0
*R. bursa*	5	0	0	0	0	0
Total	40	1 (2.5%, 0–14.0%)	0	0	0	0

## Data Availability

The sequences from this study are available in the NCBI GenBank under accession numbers PP431024-PP431074 and OR192576.

## Supplementary Materials

The following supporting information can be downloaded at https://www.mdpi.com/article/10.3390/microorganisms12071293/s1. Table S1: Tick pools collected from the vegetation of Karachay-Cherkessia in 2020. Figure S1: The map of tick collection localities in Karachay-Cherkessia. Ticks collected from patients (red circles) and vegetation (blue circles). List of collection zones: (1) Cherkessk, (2) Karachayevsk, (3) Belaya Gora, (4) Ikon-Khalk, (5) Kamennomost, (6) Kart-Dzhurt, (7) Kubina, (8) Kumysh, (9) Novaya Dzheguta, (10) Novaya Teberda, (11) Novyi Karachai, (12) Psyzh, (13) Ust-Dzheguta, (14) Zarechnyi, (15) Malokurgannyi, (16) Moskovskii, (17) Oktyabrskii, (18) Arkhyz, (19) Druzhba, (20) Znamenka, (21) Kosta Khetagurova, (22) Krasnyi Kurgan, (23) Nikolaevskoe, (24) Pervomaiskoe, (25) Schastlivoe, (26) Rim-Gorskiy, (27) Tereze, (28) Uchkeken, (29) Kholodnorodnikovskoe, (30) Chapayevskoye, (31) Prigorodnoe, (32) Kardonikskaya, (33) Krasnogorskaya, (34) Storozhevaya, (35) Frolovskii, (36) Pristan, (37) Vodorazdelnyi, (38) Michurinskii, (39) Maiskii, (40) Erken-Shakhar, (41) Adyge-Khabl, (42) Oktyabrskii, (43) Cherkessk (Green Island Park), (44) Psyzh, (45) Cherkessk (Cemetery), (46) Kavkazskii, (47) Sadovoe, (48) Znamenka, (49) Pristan, (50) Nikolaevskoe, (51) Ikon-Khalk, (52) Khabez, (53) Ali-Berdukovskii, (54) Eltarkach, (55) Chapayevskoye, (56) Vazhnoe.
