# Supplementary material for: The Diversity of Spotted Fever Group Rickettsia Found in Ixodidae Hard Ticks Removed from Humans in Karachay-Cherkessia, North Caucasus, Russia
Source: Microorganisms. 2024 Jun 25;12(7):1293. doi: 10.3390/microorganisms12071293 (PMC11278653; doi:10.3390/microorganisms12071293)
Supplement: Supplementary file 1 [file microorganisms-12-01293-s001.zip › microorganisms-2996965-supplementary.pdf]

Supplement materials

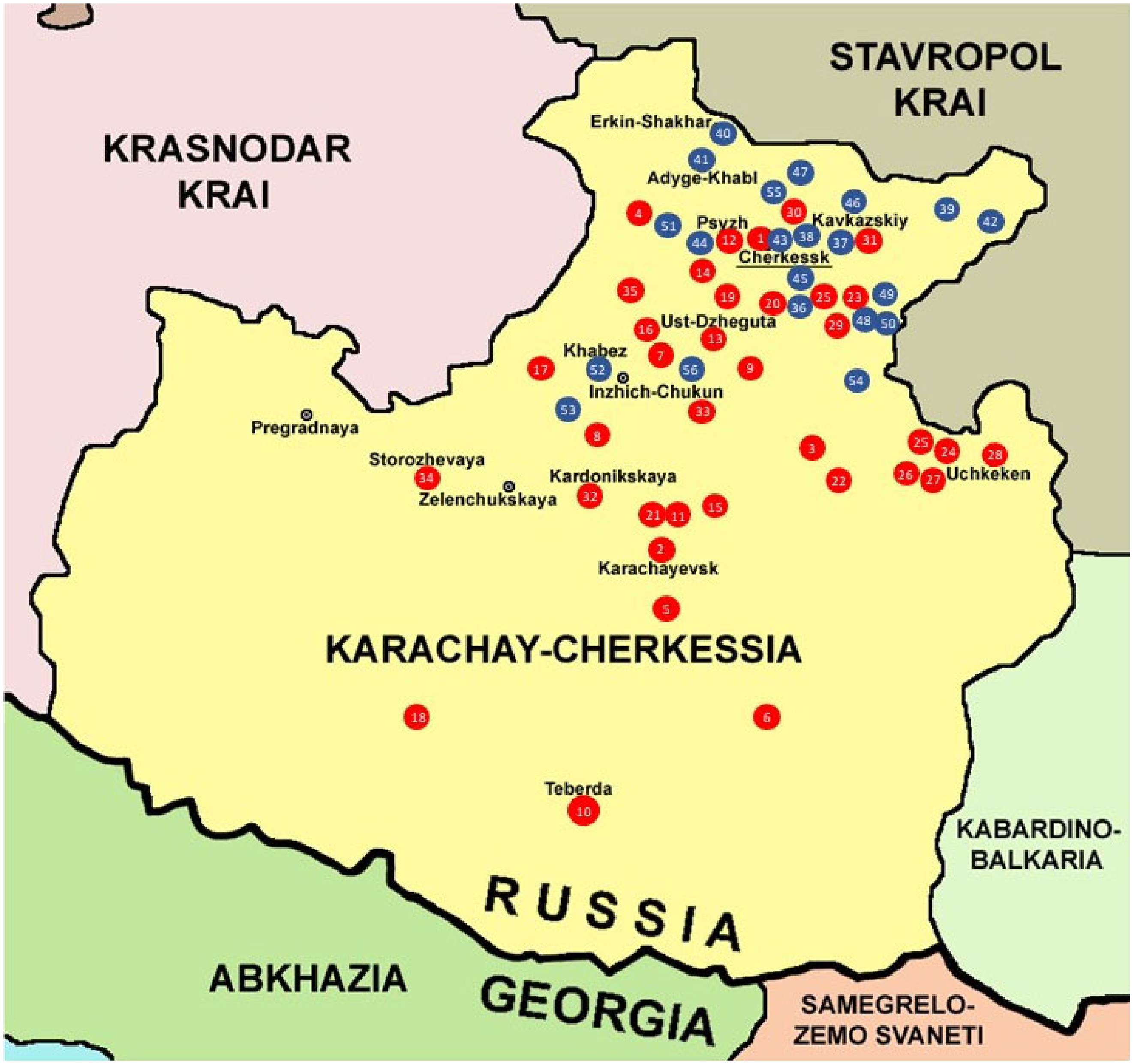

Figure S1. The map of tick collection localities in Karachay-Cherkessia. Ticks collected from patients (red circles) and vegetation (blue circles). List of collection zones: (1) Cherkessk, (2) Karachayevsk, (3) Belaya Gora, (4) Ikon-Khalk, (5) Kamennomost, (6) Kart-Dzhurt, (7) Kubina, (8) Kumysh, (9) Novaya Dzheguta, (10) Novaya Teberda; (11) Novyi Karachai; (12) Psyzh, (13) Ust-Dzheguta, (14) Zarechnyi, (15) Malokurgannyi, (16) Moskovskii, (17) Oktyabrskii, (18) Arkhyz, (19) Druzhba, (20) Znamenka, (21) Kosta Khetagurova, (22) Krasnyi Kurgan, (23) Nikolaevskoe, (24) Pervomaiskoe, (25) Schastlivoe, (26) Rim-Gorskiy, (27) Tereze, (28) Uchkeken, (29) Kholodnorodnikovskoe, (30) Chapayevskoye, (31) Prigorodnoe, (32) Kardonikskaya, (33) Krasnogorskaya, (34) Storozhevaya, (35) Frolovskii, (36) Pristan, (37) Vodorazdelnyi, (38) Michurinskii, (39) Maiskii, (40) Erken-Shakhar, (41) Adyge-Khabl, (42) Oktyabrskii, (43) Cherkessk (Green Island Park), (44) Psyzh, (45) Cherkessk (Cemetery), (46) Kavkazskii, (47) Sadovoe, (48) Znamenka, (49) Pristan, (50) Nikolaevskoe, (51) Ikon-Khalk, (52) Khabez, (53) Ali-Berdukovskii, (54) Eltarkach, (55) Chapayevskoye, (56) Vazhnoe.

Table S1. Tick pools collected from the vegetation of Karachay-Cherkessia in 2020.

Number of tick (pool)	Date	Location	Sex	Number of ticks in pool	Tick species
213	09.04.2020	Prikubansky District: Znamenka	Male	1	*Dermacentor marginatus*
214			Female	3	
215	10.04.2020	Prikubansky District: Pristan	Male	2	*Dermacentor marginatus*
216			Female	4	
217	10.04.2020	Prikubansky District: Nikolaevskoe	Female	9	*Dermacentor marginatus*
218	08.04.2020	Nogaysky District: Ikon-Khalk	Female	1	*Dermacentor marginatus*
219	08.04.2020	Khabezsky District: Khabez	Male	1	*Dermacentor marginatus*
220			Female	3	
221			Female	2	*Ripicephalus bursa*
222	09.04.2020	Prikubansky District: Maiskii	Male	9	*Dermacentor marginatus*
223			Female	5	
224	09.04.2020	Prikubansky District: Michurinskii	Female	1	*Dermacentor marginatus*
225	09.04.2020	Prikubansky District: Maiskii	Female	1	*Dermacentor marginatus*
226			Female	1	
227	09.04.2020	Prikubansky District: Kavkazskii	Female	2	*Dermacentor marginatus*
228	09.04.2020	Khabezsky District: Ali-Berdukovskii	Female	1	*Ripicephalus bursa*
229			Male	1	
230	09.04.2020	Cherkessk (Green Island Park)	Female	2	*Dermacentor marginatus*
231	09.04.2020	Prikubansky District: Vodorazdelnyi	Female	17	*Dermacentor marginatus*
232			Male	8	
233	09.04.2020	Prikubansky District: Oktyabrskii	Female	9	*Dermacentor marginatus*
234			Male	10	
235	19.04.2020	Ust-Dzhegutsky District: Eltarkach	Female	26	*Dermacentor marginatus*
236			Male	16	
237			Female	1	*Ixodes ricinus*
238	06.04.2020	Cherkessk	Male	1	*Dermacentor marginatus*
239			Female	2	*Ripicephalus bursa*
240			Female	1	*Dermacentor marginatus*
241	07.04.2020	Prikubansky District: Chapayevskoye	Female	14	*Dermacentor marginatus*
242			Male	8	
243	10.04.2020	Ust-Dzhegutsky District: Vazhnoe	Female	2	*Dermacentor marginatus*
244			Male	2	
245			Female	1	*Ripicephalus bursa*
246	08.04.2020	Abazinsky District: Psyzh	Male	1	*Dermacentor marginatus*
247	10.04.2020	Adyge-Khablsky District: Adyge-Khabl	Female	4	*Dermacentor marginatus*
248			Male	1	
249			Female	40	
250			Male	10	
251	07.04.2020	Cherkessk (Cemetery)	Female	22	*Dermacentor marginatus*
252			Male	10	
